# Ultra-high brightness Micro-LEDs with wafer-scale uniform GaN-on-silicon epilayers

**DOI:** 10.1038/s41377-024-01639-3

**Published:** 2024-10-09

**Authors:** Haifeng Wu, Xiao Lin, Qin Shuai, Youliang Zhu, Yi Fu, Xiaoqin Liao, Yazhou Wang, Yizhe Wang, Chaowei Cheng, Yong Liu, Lei Sun, Xinyi Luo, Xiaoli Zhu, Liancheng Wang, Ziwei Li, Xiao Wang, Dong Li, Anlian Pan

**Affiliations:** 1 Key Laboratory for Micro-Nano Physics and Technology of Hunan Province, State Key Laboratory of Chemo/Biosensing and Chemometrics, Hunan Institute of Optoelectronic Integration, College of Materials Science and Engineering, School of Physics and Electronics, Hunan University, 410082 Changsha, China; 2 Innovision Technology (Suzhou) Co. Ltd, 215000 Suzhou, China; 3 Lattice Power (Jiangxi) Corp., 330029 Nanchang, China; 4 College of Mechanical and Electrical Engineering, Central South University, 410083 Changsha, China; 5 Beijing Digital Optical Device IC Design Co. Ltd, 100015 Beijing, China; 6 School of Physics and Electronics, Hunan Normal University, 410081 Changsha, China

**Keywords:** Inorganic LEDs, Micro-optics

## Abstract

Owing to high pixel density and brightness, gallium nitride (GaN) based micro-light-emitting diodes (Micro-LEDs) are considered revolutionary display technology and have important application prospects in the fields of micro-display and virtual display. However, Micro-LEDs with pixel sizes smaller than 10 μm still encounter technical challenges such as sidewall damage and limited light extraction efficiency, resulting in reduced luminous efficiency and severe brightness non-uniformity. Here, we reported high-brightness green Micro-displays with a 5 μm pixel utilizing high-quality GaN-on-Si epilayers. Four-inch wafer-scale uniform green GaN epilayer is first grown on silicon substrate, which possesses a low dislocation density of 5.25 × 10^8^ cm^−^^2^, small wafer bowing of 16.7 μm, and high wavelength uniformity (standard deviation STDEV < 1 nm), scalable to 6-inch sizes. Based on the high-quality GaN epilayers, green Micro-LEDs with 5 μm pixel sizes are designed with vertical non-alignment bonding technology. An atomic sidewall passivation method combined with wet treatment successfully addressed the Micro-LED sidewall damages and steadily produced nano-scale surface textures on the pixel top, which unlocked the internal quantum efficiency of the high-quality green GaN-on-Si epi-wafer. Ultra-high brightness exceeding 10^7^ cd/m^2^ (nits) is thus achieved in the green Micro-LEDs, marking the highest reported results. Furthermore, integration of Micro-LEDs with Si-based CMOS circuits enables the realization of green Micro-LED displays with resolution up to 1080 × 780, realizing high-definition playback of movies and images. This work lays the foundation for the mass production of high-brightness Micro-LED displays on large-size GaN-on-Si epi-wafers.

## Abstract (web summary)

Ultra-high brightness green Micro-LEDs with 5 μm pixels were achieved using uniform GaN-on-Si epilayers, exceeding 10^7^ cd/m^2^ brightness and enabling high-resolution Micro-LED displays with CMOS circuit integration.

## Graphical abstract

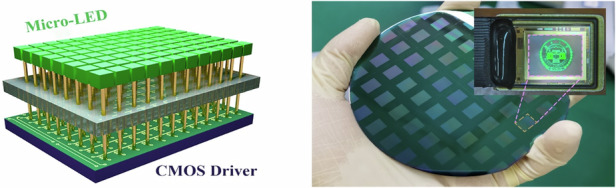

## Introduction

In recent years, the field of display technology has witnessed a paradigm shift toward Micro-LEDs displays, representing a promising avenue for next-generation visual interfaces^1,2^. Micro-LEDs offer superior brightness, contrast ratio and efficiency compared to traditional display technologies such as liquid crystal displays (LCDs) and organic light-emitting diodes (OLEDs)^3,4^. Moreover, Micro-LEDs boast longer lifespans, enhanced durability, and reduced power consumption, making them an attractive candidate for the next generation of display devices^1,5^. Among the various colors crucial for achieving vibrant and energy-efficient displays, green stands out as particularly significant due to its essential role in accurate color reproduction and overall image quality^6^. The significance of the green color extends beyond aesthetic appeal, impacting the overall visual experience, especially in applications such as high-quality imaging, augmented reality (AR), and virtual reality (VR)^7^. To achieve long-wavelength green GaN epilayers, lowering the growth temperature is a common practice to increase indium (In) incorporation^8,9^. However, growing quantum wells at lower temperatures results in nonuniform In content distribution and localized In phase separation due to insufficient atomic migration within specific growth times. This presents a formidable hurdle in achieving uniform wavelength epilayers and degrades quantum well crystal quality^10^. Additionally, the active region of GaN epilayers faces significant polarization fields as In content increases. This leads to the separation of electron and hole wave functions, reducing the radiative recombination rate and manifesting the quantum confined Stark effect (QCSE)^11,12^. Both challenges contribute to the scarcity of suitable green light source materials, commonly referred to as the “green gap”^13^. As a consequence, recent advancements in Micro-LED technology have predominantly focused on short-wavelength blue LEDs^14–16^. However, progress in green Micro-LEDs has been hindered by persistently low energy conversion efficiency, especially with small-size pixels. This limitation poses a significant obstacle in achieving a balanced color spectrum and a considerable overall brightness, crucial for delivering realistic and vibrant visuals in display technologies^17^. Hence, a detailed exploration of green Micro-LED technology becomes imperative to unlock the full potential of Micro-LED displays.

The performance of Micro-LEDs is predominantly influenced by two key factors. The first crucial aspect is the quality of materials. High crystal quality is paramount in achieving a low nonradiative recombination rate and reducing leakage current, forming the foundational basis for optimal light-emission properties^18^. Hence, advanced materials have the potential to yield brighter Micro-LEDs, thereby enhancing overall display performance^19^. Among various options, GaN on silicon substrate emerges as particularly promising for large-scale fabrication owing to its low substrate cost and well-established processing technology^20,21^. However, challenges arise from the substantial lattice mismatch (16.9%) and thermal mismatch (57%) between GaN and Si substrates, surpassing those of GaN/sapphire and GaN/6H-SiC. This dissonance can lead to issues such as high dislocation density, film cracking, and wafer bowing during the epitaxial growth of GaN, significantly impacting the material quality, the quantum efficiency, and the fabrication yield^22–24^. The second critical factor is the size of individual Micro-LED pixels. Smaller Micro-LED pixel offers the advantage of high brightness, high resolution, and compact size, which are critical for near-eye display^25,26^. It shows that a 20-micron pixel-sized GaN-based Micro-LED with a screen size of 0.18-inch can achieve a brightness up to 25,000 nits at 40 A/cm^2^ ^27^. However, as the pixel of Micro-LEDs continues to shrink, surface defects such as defect points, oxides, or other impurities may become more pronounced^28,29^. These defects trap the injected electrons and holes, leading to significantly degraded efficiency, brightness, and reliability. In addressing the issue of size-induced defects and nonradiative recombination in Micro-LEDs, various strategies have been proposed. For instance, Matthew S. Wong et al. investigated the size-dependent efficiency reduction in Micro-LEDs and found that it can be mitigated by combining potassium hydroxide (KOH) treatment with a 50 nm thickness SiO_2_ sidewall passivation layer deposited by atomic layer deposition^30^. Similarly, K.R. Son et al. demonstrated that Micro-LEDs with a SiO_2_ passivation layer exhibit higher light output power and current density compared to those with Al_2_O_3_ and Si_3_N_4_ dielectric materials. This improvement can be attributed to the diffusion of oxygen (O) atoms into the SiO_2_ passivation material, forming the Ga-O bonds at the interface^31^. In another study, Hang et al. designed a new Micro-LED chip structure featuring a resistive ITO/p-GaN junction by selectively etching the periphery of the p-GaN layer, effectively preventing holes from being injected into sidewall and thus suppressing Shockley-Read-Hall (SRH) nonradiative recombination^32^. Additionally, Liu et al. developed an etching-free pixel definition process using selective thermal oxidation (STO) method, achieving a 10-μm pixel size green Micro-LED with an on-wafer EQE of 6.48%^33^. However, tiny micro-LED pixels still suffer from poor light extraction efficiency because the conventional surface roughening process often generates micron-scale patterns that cannot be applied with a pixel size lower than 10 μm.

In this work, we present the successful development of remarkably bright green Micro-LEDs with an exceptional brightness exceeding 10^7^ cd/m² (nits). The foundation of this achievement lies in the fabrication of a wafer-scale uniform green GaN epilayer wafer on a 4-inch/6-inch Si (111) substrate. This epilayer exhibits a low dislocation density of 5.25 × 10^8^ cm^−^^2^, minimal wafer bowing (16.7 μm), and high wavelength uniformity (STDEV < 1 nm). We employed vertical non-alignment bonding techniques for Micro-LED chip construction. The processes involved wafer-level lithography, etching, and coating, contributing to the realization of the high-performance Micro-LED display. An atomic sidewall passivation method, utilizing KOH to remove residual materials and Al_2_O_3_ to repair the defects, successfully addressed the sidewall damage caused by the mesa etching process. Photoluminescence (PL) characterization indicates that the nonradiative carrier recombination at the defect sites can be effectively reduced, leading to a significant enhancement in light emission. A 30 × 30 Micro-LED array, with pixel and pitch sizes of 5 and 7.5 μm, respectively, exhibits brightness exceeding 10^7^ cd/m^2^ at a driving current density of 1000 A/cm^2^, which is among the highest in the reported results. A 0.39-inch Micro-LED display is further fabricated and integrated with Si-based CMOS circuits, demonstrating high-definition playback of movies and images. This work overcomes the bottleneck of sidewall defect problems in green Micro-LED, paving the way for micro-display applications in the real world.

## Results

### High-quality 4-inch green GaN-on-Si epilayers

**Fig. 1:**
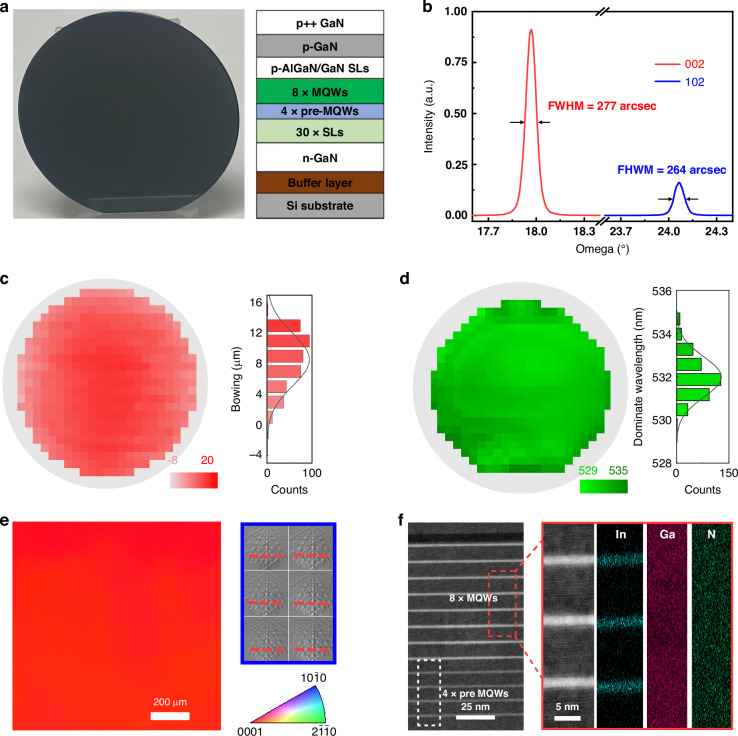
Epitaxial of InGaN/GaN quantum wells on silicon substrate for green Micro-LED chips. **a** Typical photograph of a 4-inch Si-based epitaxial wafer, where the epitaxial structure is shown on the right side. **b** High-resolution X-ray diffraction pattern of epilayer grown on Si (111) substrate, where FWHM values of rocking curves corresponding to (002) and (102) are measured to be 277 arcsec and 264 arcsec, respectively. **c** The mapping of wafer bowing condition, which indicates convex bowing of 16.7 μm. **d** Wafer mapping of the dominant wavelength on the epilayer. **e** EBSD IPF mapping and EBSP of the epilayer across a 1 × 1 mm area. **f** High-resolution transmission electron microscope (HRTEM) image and element distribution of the GaN/InGaN MQW

The green Micro-LED wafer in this work is grown on Si (111) substrate by metal-organic chemical vapor deposition (MOCVD) and a typical optical image is depicted in Fig. 1a. The epitaxy structure in the vertical direction is schematically illustrated in the right panel of Fig. 1a. The growth process is typically initiated by a 200 nm thick AlN nucleation layer, followed by 1.5 μm thick AlGaN-GaN strain-control and dislocation-annihilation stacks. It should be noted here that the growth of high-quality AlN is critical for achieving high-quality GaN-on-Si epilayers. It is well known that GaN epilayers grown on silicon substrates are prone to cracking due to the large difference in their thermal expansion coefficients. AlN nucleation layer buffers are commonly used to suppress GaN-on-Si epi cracks. However, achieving high quality typically requires growth temperatures exceeding 1400 °C due to the strong Al–N bonds. This poses a challenge since the melting point of silicon is just 1414 °C, making such high temperatures impractical on silicon substrates. To address this issue, in our work, gallium (Ga) was employed as surfactant during AlN growth. This approach promoted the migration of aluminum (Al) adatoms at a more moderate growth temperature of 1100 °C by passivating the growth surface, thereby ensuring the subsequent growth of high-quality epilayers. Following the buffer layer (AlN nucleation layer and AlGaN-GaN strain-control and dislocation-annihilation stacks), a 2 μm thick n-type GaN is grown, followed by 30 periods of InGaN/GaN superlattices and a 4×InGaN pre-quantum wells (pre-QWs, with In content of 10%) to effectively relax the compressive strain before the growth of 8 periods of green InGaN/GaN (2.5 nm/12 nm, with In content of 25%) multiple quantum wells (MQWs). P-type AlGaN/GaN superlattices were further grown and used as the electron blocking layer (EBL), followed by p-type GaN and a highly magnesium-doped contact layer to achieve a low and uniform contact resistance. High-resolution X-ray diffraction is used to characterize the crystal quality of the epilayer on Si substrate. As shown in Fig. 1b, the full width at half maximum (FWHM) values of the (002)/(102) reflection rocking curves were 277 arcsec and 264 arcsec, respectively, corresponding to a total dislocation density of ~5.25 × 10^8^ cm^−^^2^ (Supplementary Table S1). The mapping of wafer bowing condition is presented in Fig. 1c. Owning to the meticulously engineered strain-control stacks, the wafer exhibits a convex bowing of 16.7 μm at room temperature. The small wafer bow rendered it highly compatible with subsequent standard wafer-scale fabrication processes such as wafer bonding and photolithography. A wafer mapping of the dominant wavelength on the epilayer is presented in Fig. 1d, revealing an average dominant wavelength of 531.98 nm with standard deviation of 0.939 nm. Notably, the wavelength variation across the wafer was 6 nm and reduced to only 1.7 nm at the 0.39-inch scale (Fig. S1).

To ascertain the single-crystalline quality of the GaN films, electron backscattered diffraction (EBSD) is conducted on a large length scale. The inverse pole figure (IPF) map along the normal direction, associated with the [0001] crystal orientation, exhibits a consistently uniform red color, signifying identical out-plane orientation, with no discernible boundaries or rotations of domains (Fig. 1e). Moreover, examination of electron backscattered patterns (EBSPs) collected at six different positions across a 1 × 1 mm area reveals only one set of Kikuchi patterns (right panel in Fig. 1e). This unequivocal evidence establishes a singular crystal orientation throughout the entirety of the GaN films, affirming their consistent single-crystalline nature over a millimeter-scale range. This assurance of uniformity is pivotal in ensuring the precision required for designing Micro-LEDs. We also examined the distribution of indium in the quantum wells and the interface quality. As shown in Fig. 1f, we observed a clear, sharp contrast at the InGaN/GaN interface and a well-defined periodic distribution of indium, which unequivocally confirms the high quality of the epilayer materials^34,35^. This can be attributed to the incorporation of pre-QWs with low indium content, which plays a crucial role in enhancing the quality and uniformity of MQWs with higher indium concentrations, especially for green light emission. Fluorescence microscope image, Atomic Force Microscope image, and HRXRD ω-2*θ* scan results all confirm the high crystalline quality of the epilayer (Fig. S2a–c). A comprehensive analysis of material parameters, comparing our epilayer with reported GaN epilayers, is systematically presented in Supplementary Table S1 and S2. The data underscores superior performances in dislocation density and wafer bowing in our work compared to previously reported results^36,37^.

When such GaN-on-Si epi-wafers were processed to 26 × 26 mil^2^ (660 × 660 μm^2^) vertical LED power chips, they delivered a median external quantum efficiency of 45% at the current density of 30 A/cm^2^ (Fig. S2d), on par with the same size commercial vertical green LED chips grown on patterned sapphire substrates^38^. The green GaN epilayers grown on silicon substrates, with good wavelength uniformity, small wafer bowing, and decent dislocation density, provided exceptional suitability for advanced small-sized Micro-LED fabrication. Moreover, this approach is also applicable to the growth of 6-inch GaN-on-Si epilayers with low wafer bowing (Fig. S3).

### High brightness Micro-LED chips

**Fig. 2:**
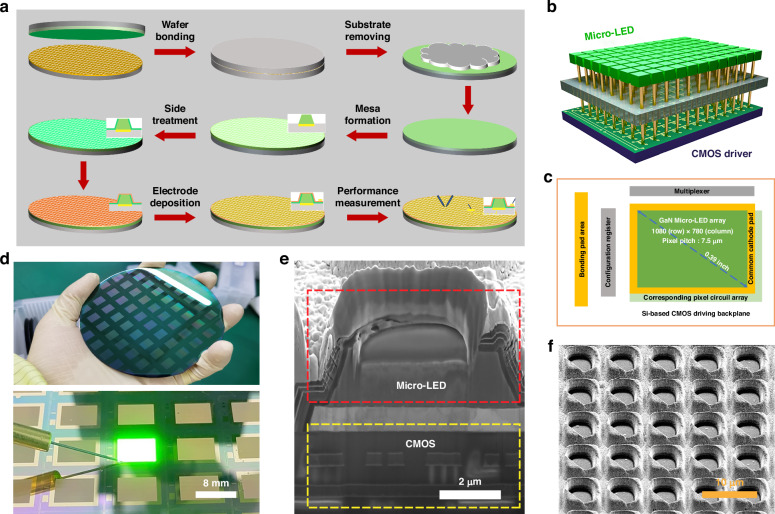
Fabrication of green Micro-LED display chips. **a** Fabrication process of the Micro-LED, involving wafer bonding, mesa formation and device fabrication process. **b** Schematic diagram of 3D integrated Micro-LED chip matrix structure. **c** Layout of the Micro-LED display. **d** Photograph of a 4-inch wafer that integrates multiply 0.39-inch green Micro-LED displays. **e** Sectional view of a pixel of the Micro-LED chips. **f** Real three-dimensional structure of the Micro-LED chips

Figure 2a presents the fabrication process of the CMOS-integrated Micro-LED chips, which involves nine main steps. Briefly, the process begins with the preparation of a pre-fabricated Si-based epitaxial wafer. Following this, a 110 nm layer of ITO is deposited, followed by the sequential deposition of 700 nm thick Cr/Pt/Au metal stacks. The wafer is then bonded to a CMOS chip without requiring any photolithography alignment process, namely non-alignment bonding process. This approach overcomes the alignment accuracy limitations typically associated with thermocompression bonding equipment, enabling the fabrication of higher resolution Micro-LED chips^14,15^. After bonding, the Si substrate is removed with an alkaline mixed solution. Device pixelation (pixel size and pitch: 5 μm and 7.5 μm) is then achieved through three-step etching processes. This includes a two-step plasma etching process, where Cl_2_ and BCl_3_ are used to remove the entire buffer layer, followed by etching the remaining GaN-based layers to expose bonding metal with a 300 nm SiO_2_ hard mask deposited by plasma chemical vapor deposition. Finally, ion beam etching system is employed to remove the metal stack with argon. To optimize the pixels and repair defects, a KOH treatment is applied due to its anisotropic effects on the sidewalls and the luminous countertop of the pixel mesas^39^. This treatment not only removes residual materials from the sidewalls but also roughens the GaN grains on the mesa surface, facilitating photon escape from the chip. Subsequently, a 50 nm Al_2_O_3_ passivation layer is applied to cover the surface, further enhancing defect repair. After opening the window to expose the n-GaN layer, cathode electrodes are established using lithography and metal evaporation techniques to finalize device fabrication and establish interconnections. More details can be found in the “Materials and methods” section. The sidewall treatment process, which is pivotal in enhancing device performances, will be discussed in detail in the later section. As a result, these Micro-LEDs can be easily manufactured and integrated on a large scale as required. Figure 2b depicts a schematic diagram of a three-dimensional (3D) integrated micro-LED chip matrix structure, where the top Micro-LED chips are driven through bottom Si CMOS circuits. Figure 2c depicts the layout of the Micro-LED display, involving Micro-LED arrays (1080 × 780), pixel circuit arrays, and peripheral circuits. Figure 2d shows a 4-inch wafer that integrated multiple Micro-LED chips with a display size of 0.39 inches, where each chip can be illuminated independently (top panel: wafer after mesa formation; bottom panel: actual picture of the chip being lit up). Figure 2e shows the sectional view of a pixel of the Micro-LED chips, where the Micro-LED is bonded to the CMOS driver tightly without any cracks, which would effectively increase brightness uniformity and reliability as compared with that achieved with the flip-chip bonding method^14,40^. The scanning electron microscope (SEM) image shown in Fig. 2f indicates the real three-dimensional structure of the Micro-LED chips. Pixels are isolated from each other to suppress sidewall light emission and minimize the light emission angle, effectively preventing luminous crosstalk by the Finite Difference Time Domain (FDTD) simulation (Fig. S4).

**Fig. 3:**
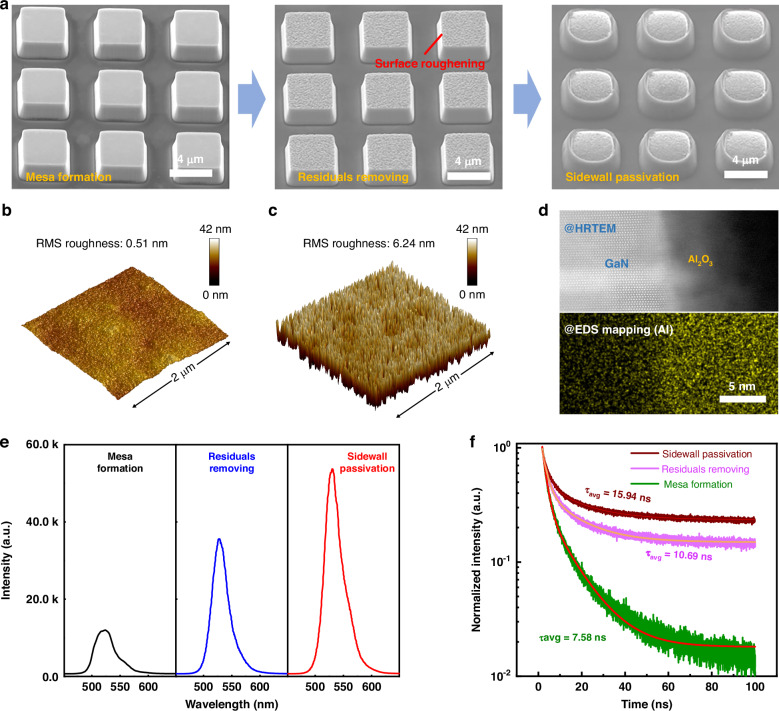
Atomic sidewall repairing with KOH and ALD treatment. **a** SEM image of the mesas (left) without any treatment, (middle) after KOH treatment and (right) after defect passivation. **b**, **c** AFM images of the mesa without any treatment (**b**) and KOH treatment (**c**). **d** Cross-sectional HRTEM and EDS images of the sidewall after Al_2_O_3_ deposition. **e**, **f** PL results (**e**) and PL decay traces (**f**) corresponding to each step shown in (**a**)

The SEM images of sidewall and surface treatment processes are elaborated in Fig. 3a. As illustrated in Fig. 3a (left), the mesas have particles and defects at the sidewall after the etching process with Inductively Coupled Plasma (ICP) equipped with Cl-based gas, which can be attributed to the following factors. On the one hand, ICP etching typically uses chemical vapor species and plasma to remove material, which can lead to chemical reactions and the formation of by-products^41^. These by-products may remain on the surface of the wafer and the sidewall of the mesas in the form of particles. On the other hand, ICP etching may not remove material uniformly, resulting in unevenness on the mesa sidewalls, which may introduce defects in the manufacturing process and create undesired nonradiative recombination centers. To address this challenge, KOH treatment is first employed to treat the non-polar sidewalls of the mesas. This process effectively eliminates residual materials, including by-products, particles, and impurities generated during the etching process. As a result, smoother and cleaner sidewalls are achieved, greatly enhancing the radiative recombination efficiency of charge carriers. At the same time, the N-polar GaN Micro-LED top surface, which is susceptible to KOH etching, readily underwent surface roughening, as illustrated in the middle of Fig. 3a. The top GaN surface was featured with uniformly distributed grains with a size of ~50 nm (Fig. S5). It is worth noting that the roughening process is highly controllable where the grain sizes can be optimized to any desired values (10–200 nm) by adjusting the duration and temperature (Fig. S6). Figure 3b, c presents the 3D morphology images of the mesa both before and after KOH treatment, where the root mean square (RMS) roughness is measured to be 0.51 nm and 6.24 nm for the respective stages, confirming the effectiveness of KOH treatment on the surface morphology. This properly roughed surface facilitated light extraction from the Micro-LED pixels and played a very important role in boosting the overall external quantum efficiency of our Micro-LED display, which is validated through FDTD simulations. Figure S5 confirms that these small GaN roughened grains can effectively enhance the light extraction efficiency. Notably, the roughed grain sizes in this work are smaller than the previous report^42^, indicating better compatibility with the micron-scale Micro-LED top surface. The corresponding PL results are depicted in Fig. 3e, where the peak emission intensity shows a threefold enhancement after KOH treatment, which again proves the efficiency of this surface roughing technique.

Atomic layer deposition (ALD) process is then utilized to passivate the sidewall and repair the defects. The ALD possessed exceptionally precise control over material deposition with the accuracy down to a single molecular layer. In this work, aluminum oxide was selected as the passivation material for defect repairing with atomic-level precision, effectively filling tiny surface imperfections such as cracks, voids, or uneven regions. Figure 3d displays the HRTEM and EDS mapping images of the interface between MQWs and Al_2_O_3_, showcasing a well-covered sidewall of the MQWs with Al_2_O_3_. The PL emission of the Al_2_O_3_-passivated mesas is approximately 5 times higher than that without any treatment (Fig. 3e), demonstrating an outstanding repairing effect. The photoluminescence (PL) decay traces corresponding to each stage are illustrated in Fig. 3f. The findings reveal a notable enhancement in the average carrier recombination lifetime—from 7.58 ns to 10.69 ns following KOH treatment, ultimately reaching 15.94 ns after the passivation of mesas with Al_2_O_3_. This suggests that the atomic sidewall passivation method effectively suppresses nonradiative recombination probability by reducing defect states on the sidewall surface in Micro-LEDs^43^.

**Fig. 4:**
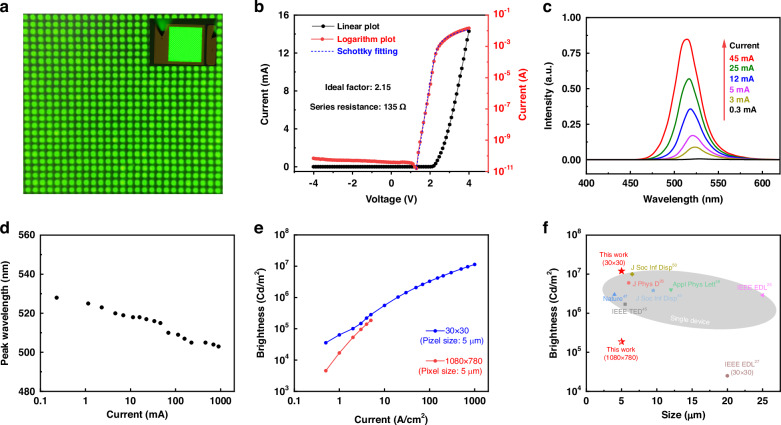
EL properties of the Micro-LED. **a** A real Micro-LED display image acquired using a probe station. **b** IV characteristics in linear and log scale. **c** EL spectra measured at the different currents. **d** The extracted peak wavelength and **e** brightness performance with different currents. **f** Comparison of Micro-LEDs’ key parameters in this work with some typical reported results

Figure 4a shows a real luminescence photograph of a 30 × 30 Micro-LED array, which can emit a uniform green light. The IV curve across the Micro-LED array is presented in Fig. 4b. It shows a typical diode rectifying behavior with rectifying ratio over 10^8^, which is higher than most of the reported devices^44^. We also fitted the IV curve with the Shockley equation and achieved an ideal factor of 2.15, indicating that the transport is dominated by the recombination of the electrons and holes at the MQWs rather than the diffusion process. The turn-on voltage is deduced to be 2.1 V from the linear plot of IV cure, which is much smaller than the reported results that are typically falls within the range of 2.5 to 2.8 volts^45,46^. The series resistance is deduced to be 135 Ω, indicating nice contact of the electrodes on both p-GaN and n-GaN, largely reduced Joule heat generation of the Micro-LEDs.

Figure 4c, d shows the electroluminescence (EL) intensity profiles and the peak wavelength of the Micro-LED obtained at different injection current. It indicates a blue shift from 530 to 505 nm while the current increases from 0.3 to 900 mA. This can be reasonably attributed to the piezoelectric polarization degraded in the MQWs and the radiative recombination of carriers at higher energy state due to high injection current^25^. 0.39-inch (1080 × 780) green Micro-LED screens are also measured. The corresponding brightness information is plotted in Fig. 4e, for the 30 × 30 Micro-LEDs array, the brightness increases with injection current and reaches a maximum of 1.2 × 10^7^ cd/m^2^ (nits) at 1000 A/cm^2^. For 0.39-inch Micro-LED screens, the brightness also shows a similar increasing trend up to 186017 Cd/m^2^ at 5 A/cm^2^. Figure S7 depicts the EQE as a function of total injection current, demonstrating a peak EQE value of 7.17% at current density of 2 A/cm^2^. Figure 4f shows the comparison of brightness parameter of our Micro-LED with some typical reported results, where our result represents one of the highest values among the reported results^25–27,45,47–50^, specific details are in Supplementary Table S3.

### Active matrix Micro-display

**Fig. 5:**
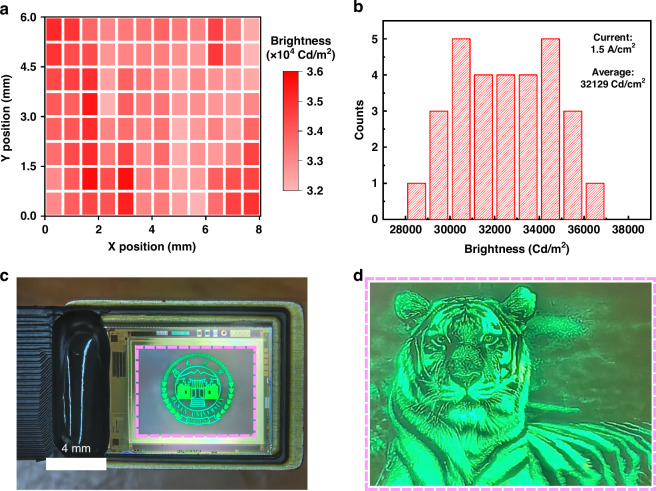
Images displayed by a CMOS driver-integrated Micro-LED screen. **a** The brightness mapping of single micro-display, indicating high uniformity with STDEV < 720 Cd/m^2^ (2.2%). **b** The statistical distribution of the brightness of 30 micro-displays on the same Epitaxial Wafer. **c** A real image of a packaged Micro-LED display chip. **d** Real display images of tiger displayed by the display screen

Figure 5a illustrates the brightness mapping of a single micro-display obtained from ninety-six different points across 8 × 6 mm areas at a driving current of 1.5 A/cm². Impressively, the standard deviations (STDEV) are exceptionally small, measuring below 720 Cd/m² (2.2%), with an average brightness of 32,987 Cd/m². The brightness uniformity at higher current densities is further tested and depicted in Fig. S8. These results demonstrate that the uniformity does not deteriorate at higher current densities. When demura technology is applied, the uniformity for micro-display screen can be further improved (Fig. S9). Figure 5b showcases the brightness of 30 distinct micro-displays on the same wafer at the same driving current, revealing STDEV and average brightness values of 1365 Cd/m² (4.2%) and 32,129 Cd/m², respectively. This exceptional brightness uniformity can be attributed to the uniform GaN-on-silicon epilayers and advanced fabrication technology. Figure 5c shows a packaged Micro-LED display chip, showing the logo of Hunan University. The display has 829, 400 (1080 × 780) pixels with pitch size of 7.5 μm, corresponding to 3400 pixels per inch (PPI). Each pixel comprised a 5-μm Micro-LED and Si-based CMOS driver. All pixels shared a common cathode pad with an independently addressable scan line and data line. When a specific pixel receives a voltage *V*_scan_ on the scanning line and *V*_data_ on the data line, the pixel is illuminated. The image is maintained in a static display mode through the charging and discharging of capacitors at a refresh rate of 60 Hz. Moreover, the display possesses an 8-bit grayscale display capability, enabling contrast control when displaying images and movies. Figure 5d shows the enlarged real image of tiger displayed by the display screen, more images can be seen in Fig. S10, where every detail can be clearly resolved. It demonstrates a clear imaging capability and successfully achieves high-definition playback of images and movies (Supplementary Video 1).

## Discussion

In conclusion, we present a wafer-scale uniform 4-inch green GaN-on-silicon epilayer wafer characterized by low dislocation density, outstanding wavelength uniformity, and minimal wafer bowing. Atomic sidewall passivation approach is developed to address the issue of sidewall defects in small-sized Micro-LEDs, which effectively reduces carrier recombination at defect sites. This passivation process, together with the well-controlled surface roughing, significantly enhanced the optical and electrical performance of the green Micro-LEDs. The high-resolution, highly bright GaN-on-Si Micro-LED array with pixel sizes as small as 5 μm exhibits a brightness exceeding 10^7^ cd/m^2^ (nits). Additionally, the vertical non-alignment bonding technique is employed to seamlessly integrate the Micro-LED display with a Si-based CMOS driver. This integration not only achieves remarkable brightness uniformity but also a high-resolution image display. This work highlights the potential of Micro-LED displays and drives their widespread application in real-world scenarios.

## Materials and methods

### Growth of GaN-on-Si epilayers

A Si-based green epitaxial wafer is grown by MOCVD; high-purity nitrogen (N_2_) and hydrogen (H_2_) are used as carrier gases; trimethylgallium (TMGa) and trimethylindium (TMIn), trimethylaluminum (TMAl) and ammonia (NH_3_) are used as Ga, In, Al and N precursors; SiH_4_ and Cp_2_Mg are used as doping source of n-type and p-type GaN.

### Micro-LED chip fabrication

After epitaxial growth, the wafer is coated with ITO (110 nm) onto p++ GaN by sputter and annealed at 500 °C for 5 min by RTA, and then a 700 nm thick Cr/Pt/Au metal stack layer is deposited by E-beam evaporation, which is prepared and bonded to a support CMOS substrate. The epitaxial Si (111) substrate is then removed using alkaline mixed solution (NaOH, Isopropyl alcohol and H_2_O) at 85 °C for 30 min. High resistance buffer layer is removed with ICP (Plasmalab 133 ICP 380, Oxford). GaN mesa is achieved through photolithography with a stepper lithography machine (NSR-2205i11, Nikon) and etching processes using BCl_3_/Cl_2_ gas with ICP. KOH with a mass fraction of 18% treatment is further conducted to the GaN mesa at 40 °C; 50 nm Al_2_O_3_ was further deposited using ALD (TFS 200, Beneq). Finally, after opening the window on the top of mesa, current spreading layer (110 nm ITO) is deposited onto n-GaN layer by sputter and annealed at 300 °C for 3 min, and then a 1.5-μm thick Cr/Al/Ti/Pt/Au metal stacks layer is deposited as common cathode electrode by E-beam evaporation.

### Sample characterization

The optical images of the Micro-LED were characterized through a microscope (Zeiss Axio Scope A1) and a camera of mate 60. The SEM images were characterized by the field-emission SEM (Nova NanoSEM 230, FEI). The TEM and HRTEM images were acquired with a transmission electron microscope (FEI Themis Z (3.2)). PL spectra were measured on confocal microscope systems (WITec, Alpha 300R) equipped with an excitation of 488 nm laser.

The electrical performance of the as-fabricated devices was conducted using a self-built probe station with the spectroradiometer cs-2000a coupled with a high-accuracy luminance meter and spectrometer and a Keithley 2450 for current source. The photons were collected by the spectroradiometer cs-2000a when Micro-LEDs were given to a constant current to calculate corresponding brightness value. In order to more conveniently detect and evaluate Micro-LED brightness values, we took the luminance meter probe area as the actual area where photons are collected. It means non-illuminated areas between pixels have not been removed.

## Supplementary information

Supplementary materials for Ultra-high brightness Micro-LEDs with wafer-scale uniform GaN-on-silicon epilayers

Supplementary Video/ Movie-
