# Supplementary material for: Ultra-high brightness Micro-LEDs with wafer-scale uniform GaN-on-silicon epilayers
Source: Light Sci Appl. 2024 Oct 9;13:284. doi: 10.1038/s41377-024-01639-3 (PMC11464674; doi:10.1038/s41377-024-01639-3)
Supplement: Supplementary file 1 — Supplementary materials for Ultra-high brightness Micro-LEDs with wafer-scale uniform GaN-on-silicon epilayers [file 41377_2024_1639_MOESM1_ESM.docx]

**Supplementary Information for**

**Ultra-high brightness Micro-LEDs with wafer-scale uniform GaN-on-silicon epilayers**

Haifeng Wu^1^, Xiao Lin^1,3^, Qin Shuai^1^, Youliang Zhu^1,3^, Yi Fu^5^, Xiaoqin Liao^1^, Yazhou Wang^3^, Yizhe Wang^1^, Chaowei Cheng^4^, Yong Liu^1^, Lei Sun^6^, Xinyi Luo^1^, Xiaoli Zhu^1^, Ziwei Li^1^, Liancheng Wang^4^, Xiao Wang^1^, Dong Li^1^*, and Anlian Pan^1,2^*

^1^ Key Laboratory for Micro-Nano Physics and Technology of Hunan Province, State Key Laboratory of Chemo/Biosensing and Chemometrics, Hunan Institute of Optoelectronic Integration, College of Materials Science and Engineering, School of Physics and Electronics, Hunan University, Changsha, 410082, China

^2^ School of Physics and Electronics, Hunan Normal University, Changsha, 410081, China

^3^ Innovision Technology (Suzhou) Co., Ltd, Suzhou, 215000, China

^4^ College of Mechanical and Electrical Engineering, Central South University, Changsha, 410083, China

^5^ Lattice Power (Jiangxi) Corp., Nanchang, 330029, China

^6^ Beijing Digital Optical Device IC Design Co., Ltd, Beijing, 100015, China

^*^Corresponding Author: [liidong@hnu.edu.cn](mailto:liidong@hnu.edu.cn); [anlian.pan@hnu.edu.cn](mailto:anlian.pan@hnu.edu.cn)

**Section 1 Supplementary Figures**

**
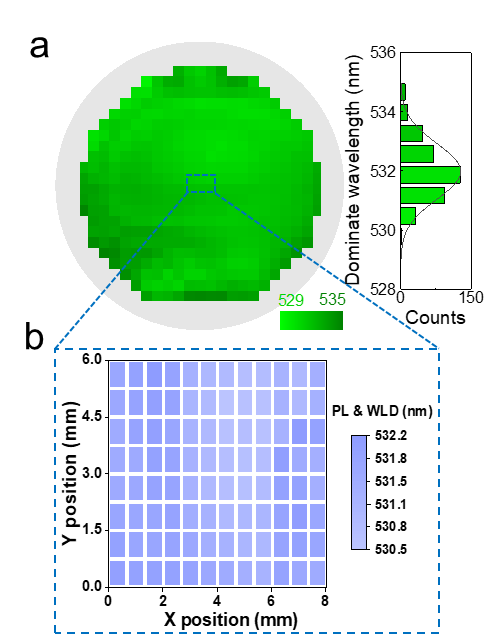
**

Fig. S1. a, The wavelength uniformity across a 4-inch wafer, with a wavelength deviation of approximately 6 nm. b, The wavelength uniformity with a randomly selected 0.39-inch region, where the shortest wavelength was 530.5 nm, and the longest was 532.2 nm, resulting in a deviation of less than 2 nm.

**
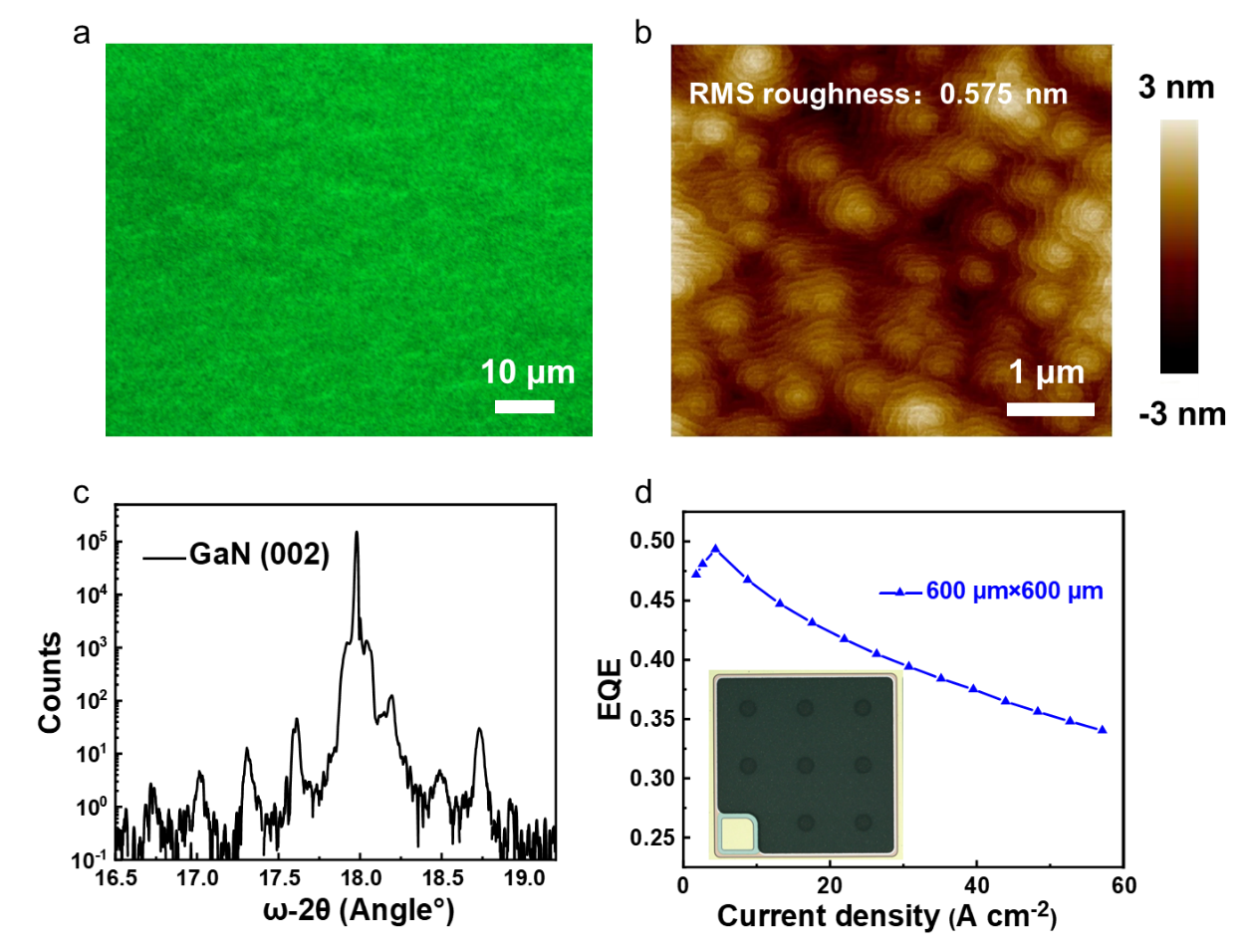
**

Fig. S2. a, Local fluorescence microscope image of a 4-inch green GaN epilayers, showcasing no indium phase separation. b, Local AFM image with a 4-inch green GaN epilayers wafer, showing an RMS roughness of 0.575 nm. c, HRXRD ω-2𝜃 scan of GaN (002) plane, where satellite peaks are clearly observed, indicating high crystal quality and sharp quantum well interface. d, EQE curve of a green GaN-based LED device with size of 600 μm×600 μm, where the peak EQE value is 49.3%.

**
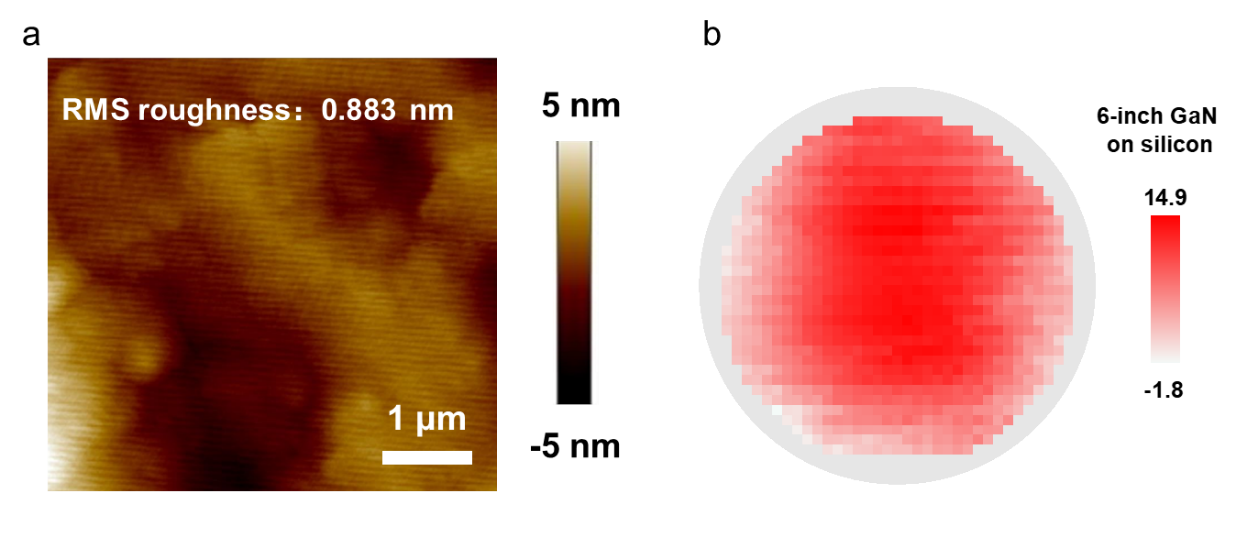
**

Fig. S3. a, AFM image of 6-inch green epilayers. The RMS roughness is 0.883nm. b, the mapping of wafer bowing condition of 6-inch green epilayers. The wafer bowing is 16.7 μm. It demonstrates that our epitaxy growth technology is full capability for the large-scale Micro-LED epilayer manufacturing process.


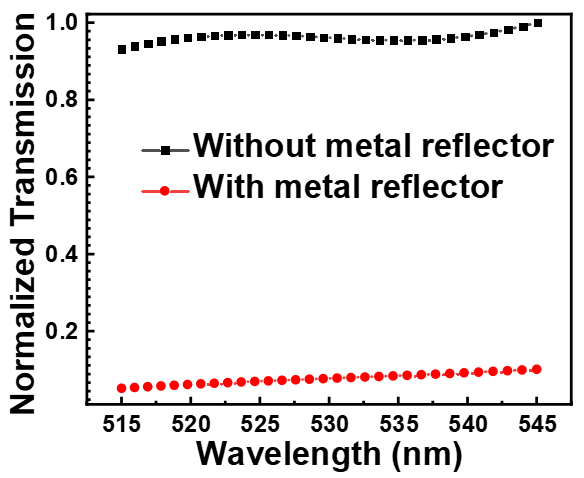


Fig. S4. The FDTD calculation of normalized transmission of mesa sidewalls. The transmission with the metal reflector is an order of magnitude lower than that without metal reflector, effectively decreasing the light emission angle and inhibiting the luminous crosstalk.


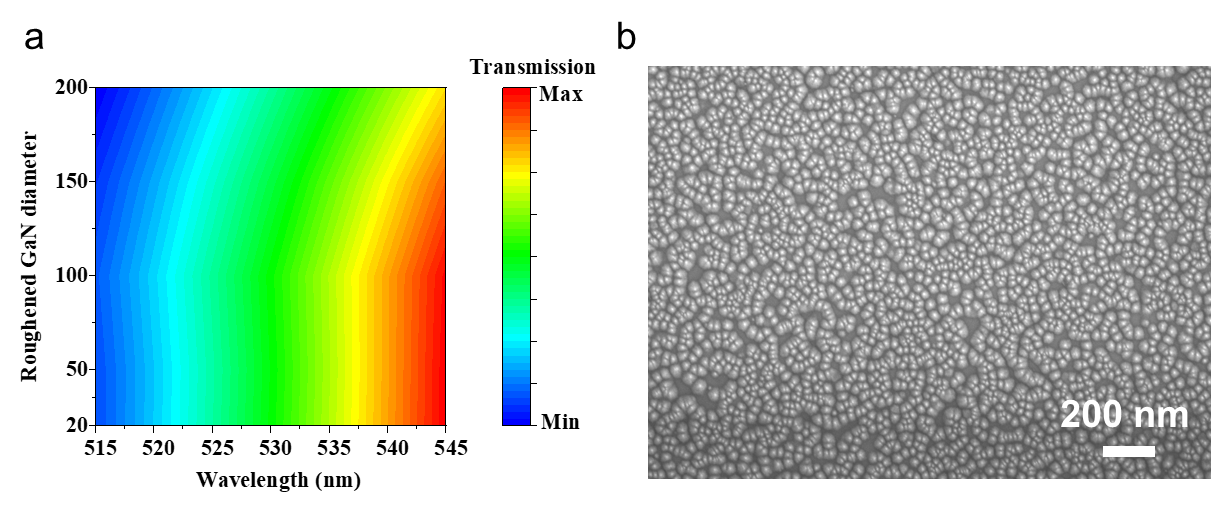


Fig. S5. a, The SEM image of roughened GaN grains. The sizes is ~ 50 nm on the top of mesas. b, The FDTD transmission calculation on mesa surface with different roughened GaN grains.


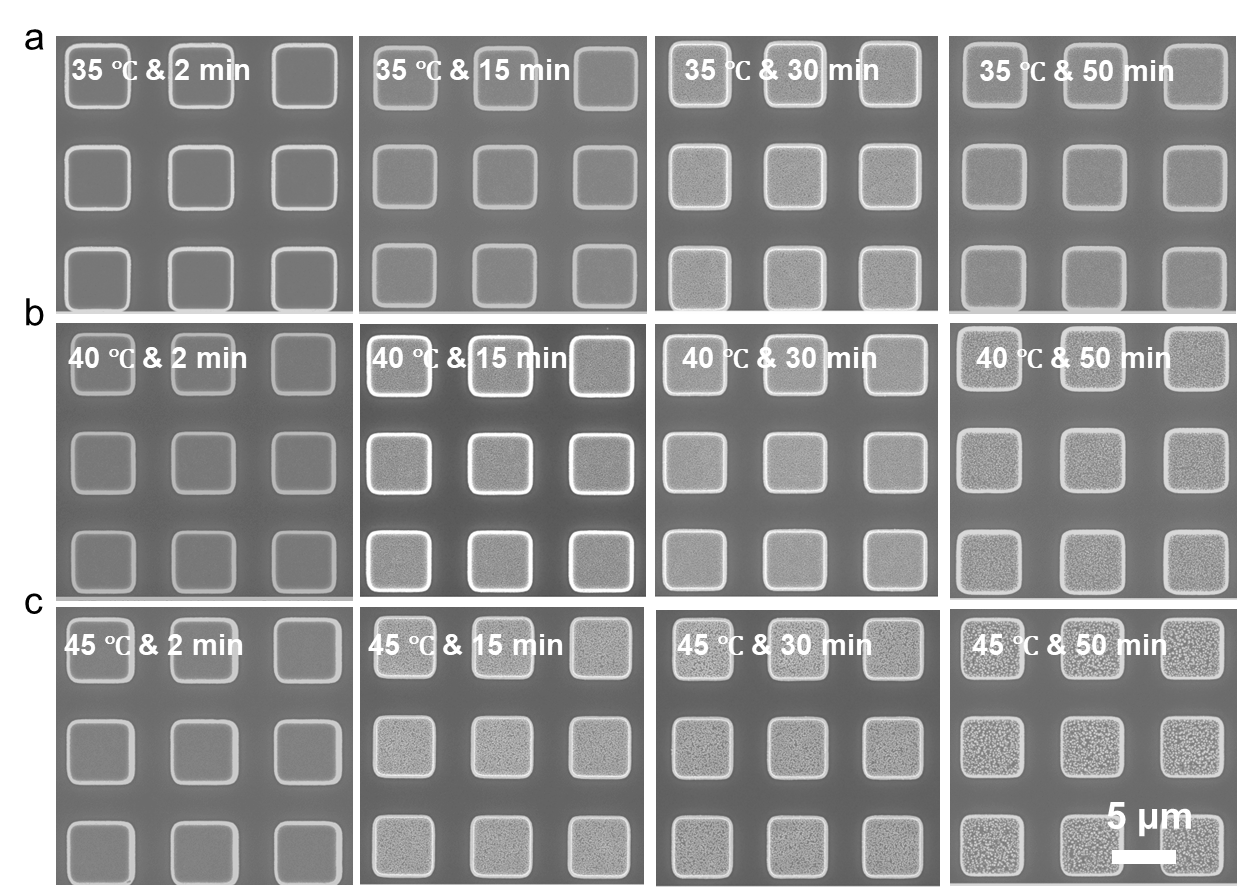


Fig. S6. a-c: The SEM images of roughening surface morphology with different conditions. By carefully controlling both temperature and duration, we achieved a range of grain sizes from 10 to 200 nm, indicating the nice controllability of the roughening process.


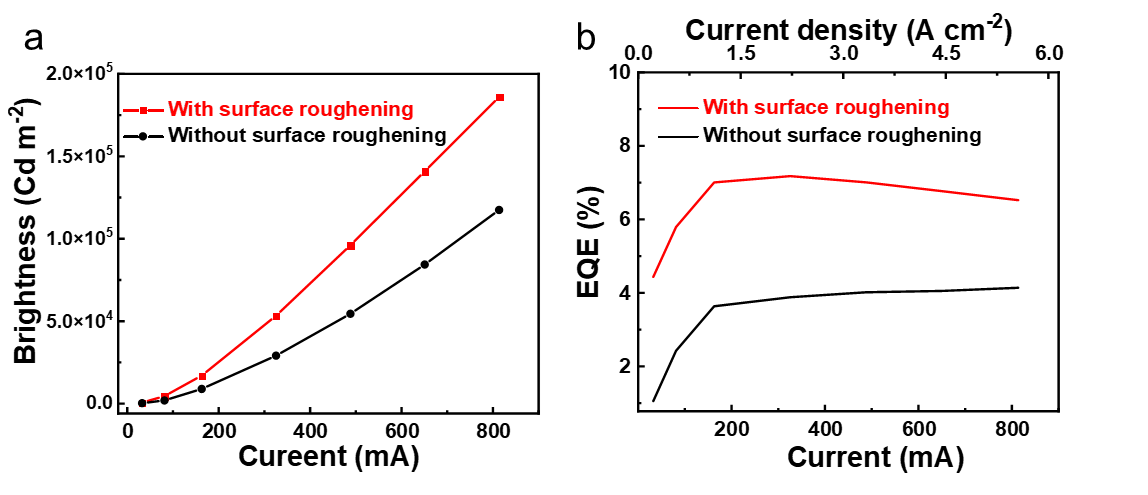


Fig. S7. a-b: Brightness and EQE as a function of total injection current, using 0.39-inch green Micro-LED display as a panel-level characterization.

A comparative analysis of brightness measurements before and after KOH treatment have been included. The brightness was measured with a self-built probe station, equiped with a spectroradiometer cs-2000a, coupled with high accuracy luminance meter and spectrometer, and driven by a Keithley 2450 current source. As shown in Fig. S7a, both samples, with and without surface roughening, exhibits a linear increase in brightness with increasing current injection. The maximum brightness values reached 186017 and 117516 Cd m^-2^, when driven by a DC current of 813 mA. Notably, at driving currents of 16 mA and 813 mA, the brightness of the roughened samples was 2.38 and 1.58 times higher than that of the non-roughened samples. This clearly demonstrates that our surface roughening process significantly enhances the efficiency of the Micro-LED chips.

An EQE analysis is crucial for a deeper understanding of the efficiency improvements.According to the definition of EQE, which represents the ratio of emitted photons to injected electrons, and assuming a non-Lambertian reflector for the light distribution emitted by the micro-LED array, the EQE values can be calculated as follow^1^:

$EQE=\frac{\pi\cdot L_{V}\cdot n\cdot p^{2}\cdot e}{h\cdot c\cdot i\cdot683\left[ lm/W \right]}\cdot\frac{\int_{380}^{780} \lambda\cdot F\left( \lambda\right)d\lambda}{\int_{380}^{780} V\left( \lambda\right)\cdot F\left( \lambda\right)d\lambda}$ (1)

Where n, p, e, h, c, i, λ, F(λ), and V (λ) are the pixel numbers, the pixel pitch size, the unit electron charge, the planck constant, the velocity of light, the lording current, the wavelength, the relative electroluminescence (EL) intensity, and the luminosity function provided by the Commission International de l’Eclairage chromaticity (CIE), respectively.

As shown in Fig. S7b, the EQE as a function of total injection current demonstrates that the EQE values are significantly higher after surface roughening process. This improvement can be attributed to that the enhanced photon escape ability facilitated by the roughened surfaces. Additionally, it is noteworthy that the sample with surface roughening exhibits a peak EQE at the current of 325 mA (2 A cm^-2^), while that of the unprocessed sample is at the higher current of 813 mA (5 A cm^-2^). This shift can be attributed to the reduction in non-radiative Shockley-Read-Hall (SRH) recombination rate, with non-radiative SRH recombination centers becoming saturated at lower currents. This suggests that the KOH treatment not only improves photon extraction but also repairs plasma etching damage at the pixel mesa sidewalls.

**
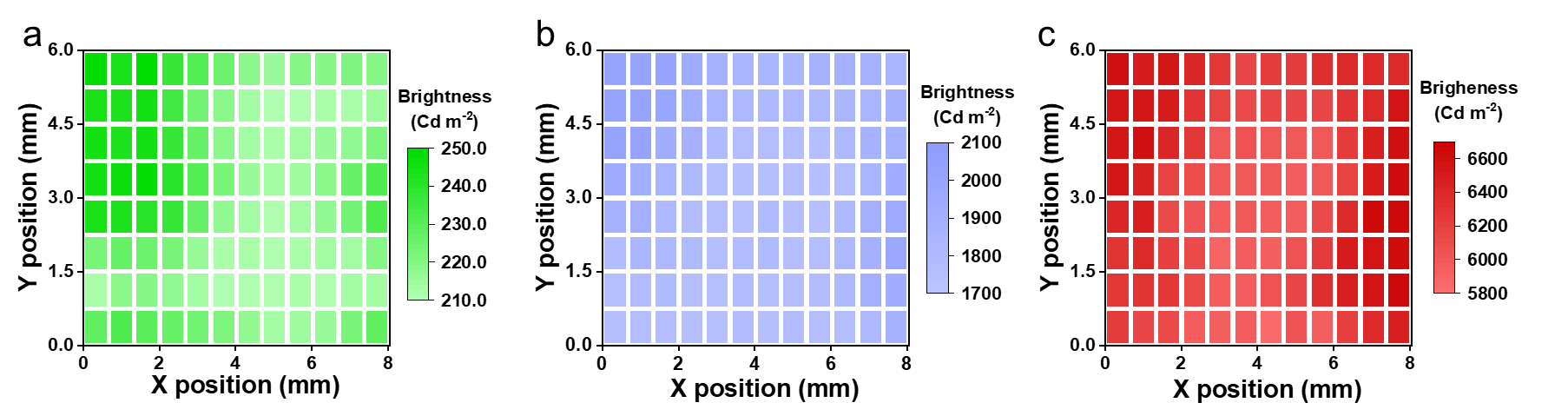
**

Fig. S8. a-c, the brightness mapping of a 0.39-inch micro-display with different current density (a: 1.5 A cm^-2^, b: 10 A cm^-2^, c: 50 A cm^-2^), where no demura technology is applied. The relative brightness uniformity with standard deviations are 11.6, 81.4, and 225 Cd m^-^², corresponding to average ratios of 5.17%, 4.43%, and 3.6%, respectively.


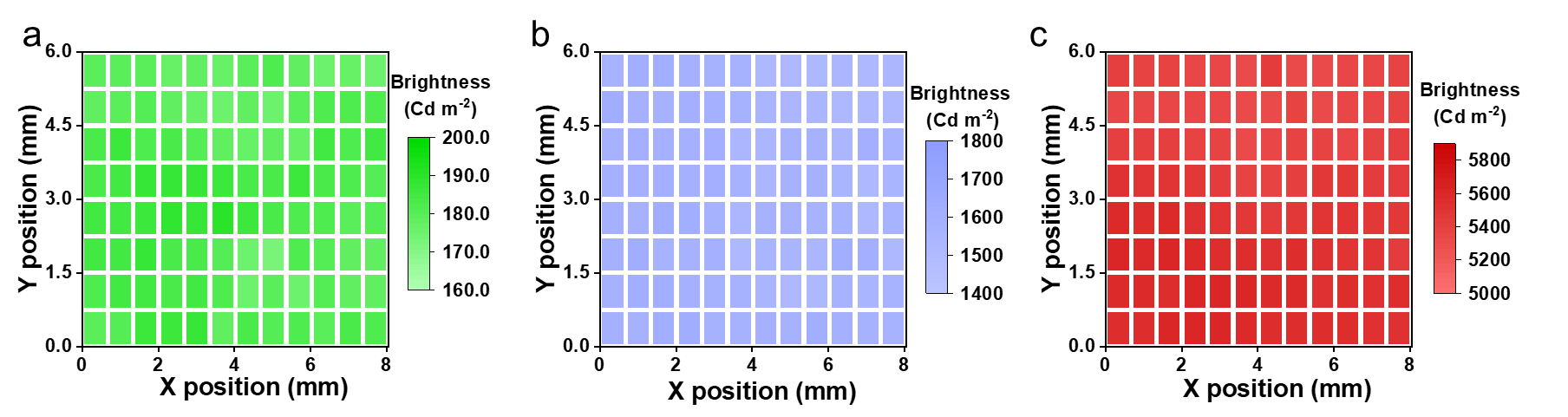


Fig. S9. a-c, the brightness mapping of a 0.39-inch micro-display with different current density (a: 1.5 A cm^-2^, b: 10 A cm^-2^, c: 50 A cm^-2^) with demura technology applied. We can observe clearly that the uniformity has improved significantly as compared with that shown in Fig. S8.


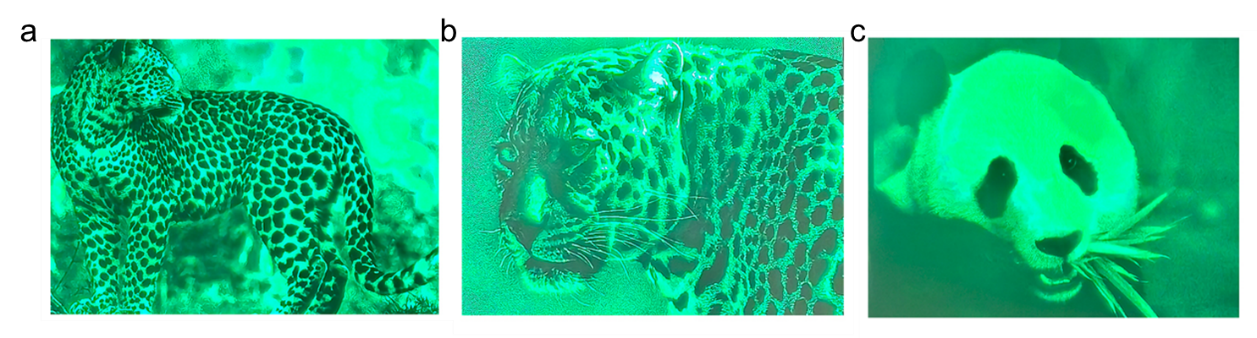


Fig. S10. a, b, c are the enlarged real images of leopard, tiger and panda displayed by the display screen, where every detail can be clearly resolved.

**Section 2 Supplementary Tables**

**Supplementary Table S1 |** the full width at half maximum (FWHM) values of the (002)/(102) reflection rocking curves and their corresponding dislocation density values.

| **Substrate** | **GaN thickness**  **(μm)** | **FWHM**  **GaN (002)** | **FWHM**  **GaN (102)** | **D_screw_**  **(cm^-2^)** | **D_edge_**  **(cm^-2^)** | **D_total_**  **(cm^-2^)** | **Ref.** |
| --- | --- | --- | --- | --- | --- | --- | --- |
| Sapphire | 6 | 322 | 272 | 2.08×10^8^ | 2.14×10^8^ | 4.22×10^8^ | 2 |
| Sapphire | 3 | 187 | 260 | 7.03×10^7^ | 3.57×10^8^ | 4.27×10^8^ | 3 |
| Si (111) | 1.2 | 317 | 432 | 2.02×10^8^ | 9.9×10^8^ | 1.19×10^9^ | 4 |
| Si (111) | 3.3 | 357 | NA | 2.56×10^8^ | NA | NA | 5 |
| Si (111) | 5.4 | 235 | 307 | 1.11×10^8^ | 5×10^8^ | 6.11×10^8^ | 6 |
| Si (111) | 6.3 | 231 | 317 | 1.07×10^8^ | 5.33×10^8^ | 6.4×10^8^ | 7 |
| Si (111) | 1.5 | 390 | 440 | 3.05×10^8^ | 1.03×10^9^ | 1.31×10^9^ | 8 |
| Si (111) | NA | 312 | 383 | 1.95×10^8^ | 7.78×10^8^ | 9.73×10^8^ | 9 |
| Si (111) | 2.5 | 337 | 353 | 2.28×10^8^ | 6.61×10^8^ | 8.89×10^8^ | 10 |
| Si (111) | 1.5 | 270 | 521 | 1.46×10^8^ | 1.44×10^9^ | 1.59×10^9^ | 11 |
| **Si (111)** | **4** | **278** | **262** | **1.55×10^8^** | **3.7×10^8^** | **5.25×10^8^** | **This Work** |

Dislocation density of screw dislocation and edge dislocation are deduced from equations

$D_{screw}=\frac{\beta_{0002}^{2}}{2\pi\ln2\times\left| b_{screw} \right|^{2}}$ (2)

$D_{edge}=\frac{\beta_{10-12}^{2}}{2\pi\ln2\times\left| b_{edge} \right|^{2}}$ (3)

where b_screw_ and b_edge_ are the Burger vectors of the screw and edge dislocations in GaN, the values are 0.4355 nm and 0.3189 nm, respectively.

**Supplementary Table S2 |** The bowing of InGaN (/AlGaN) epitaxial wafer on sapphire and Si (111) substrate.

| Substrate | Substrate size  &thickness | Epi thickness  (μm) | Epi style | Application | Bowing (μm) | Ref. |
| --- | --- | --- | --- | --- | --- | --- |
| Sapphire | 4-inch & NA | 5.8 | AlGaN | UVC-LED | ~150 | 12 |
| Sapphire | 2-inch & 430 μm | NA | InGaN | LED | ~100 | 13 |
| Sapphire | 4-inch & NA | NA | InGaN | Micro-LED | 80 | 14 |
| Sapphire | 2-inch & 430 μm | 6 | InGaN | LED | 29.5 | 15 |
| Si (111) | 8-inch & 725 μm | NA | GaN | HEMT | 21.27 | 16 |
| Si (111) | 4-inch & NA | NA | InGaN | Micro-LED | 37.06 | 17 |
| Si (111) | 8-inch & NA | 6.5 | InGaN | Micro-LED | 45 | 18 |
| Si (111) | 8-inch & NA | 6.3 | GaN | Heterojunction | 50 | 19 |
| Si (111) | 8-inch & 650 μm | 2.6 | AlGaN/GaN | Heterojunction | 18 | 20 |
| Si (111) | 8-inch & 675 μm | 3.8 | AlGaN/GaN | Heterojunction | 77.09 | 21 |
| Si (111) | **4-inch & 1500 μm** | **5.6** | **InGaN** | **Micro-LED** | **16.7** | **This Work** |
| Si (111) | **6-inch & 950 μm** | **3.2** | **InGaN** | **Micro-LED** | **16.7** |  |

**Supplementary Table S3 |** The details of green micro-LED brightness with different pixel size.

| Micro-display | Pixel size& pitch | Luminous  area | Current  (A cm^-2^) | Lv (nits) | Substrate | Ref. |
| --- | --- | --- | --- | --- | --- | --- |
| 0.39 inch | 5.5 μm & 7.5 μm | Single pixel | 1050 | 1.7×10^6^ | Sapphire | 22 |
| 0.18 inch | 20 μm & 80 μm | 32×32 | 40 | 2.5×10^4^ | Sapphire | 23 |
| RGB stacked device | 4 μm & NA | Single device | 2000 | 2×10^6^ | Sapphire | 24 |
| 0.39 inch | 12 μm & 15 μm | Single pixel | NA | 4×10^6^ | Sapphire | 25 |
| Prototype device | 6 μm & NA | Single device | 10 | 6×10^6^ | Sapphire | 26 |
| Prototype device | 25 μm & NA | Single device | 300 | 2.89×10^6^ | Sapphire | 27 |
| 0.72 inch | NA & 9.5 μm | Single pixel | NA | 3.8×10^6^ | Sapphire | 28 |
| 873 × 500 array | 6.5 μm & 10 μm | Single pixel | NA | 1×10^7^ | Sapphire | 29 |
| 30×30 array | **5 μm & 7.5 μm** | **30×30** | **1000** | **1.2×10^7^** | **Si (111)** | **This work** |
| 0.39 inch | **5 μm & 7.5 μm** | **1080×780** | **5** | **1.8×10^5^** | **Si (111)** |  |

Note:

For engineering detection of the brightness value of Micro-LEDs, we take the luminance meter probe area as the actual area where photons are collected. In other words, the non-illuminated areas between pixels are not removed. Therefore, the actual value of our Micro-LEDs’ brightness should be higher with below calculation method as single device or pixel according to other reports:

$Lv=\frac{Luminous value}{Luminous area}$ (4)

**Supplementary references**

1. Nie J. et al. Systematic study on size and temporal dependence of micro-LED arrays for display applications. *Photonics Res*, **11**: 549-557 (2023).
2. Liu, B. et al. Atomic Mechanism of Strain Alleviation and Dislocation Reduction in Highly Mismatched Remote Heteroepitaxy Using a Graphene Interlayer. *Nano Lett.* **22**, 3364-3371 (2022).
3. Tao, H. et al. Improved crystal quality and enhanced optical performance of GaN enabled by ion implantation induced high-quality nucleation. *Opt. Express* **31**, 20850-20860 (2023).
4. Lee, J.-H. & Im, K.-S. Growth of high quality GaN on Si (111) substrate by using two-step growth method for vertical power devices application. *Crystals* **11**, 234 (2021).
5. Lv, Q. et al. Realization of highly efficient InGaN green LEDs with sandwich-like multiple quantum well structure: role of enhanced interwell carrier transport. *ACS Photonics* **6**, 130-138 (2018).
6. Khadar, R. A. et al. 820-V GaN-on-Si quasi-vertical pin diodes with BFOM of 2.0 GW/cm2. *IEEE Electron Device Lett.* **39**, 401-404 (2018).
7. Zhang, Y., Yuan, M., Chowdhury, N., Cheng, K. & Palacios, T. 720-V/0.35-mΩ cm^2^ Fully Vertical GaN-on-Si Power Diodes by Selective Removal of Si Substrates and Buffer Layers. *IEEE Electron Device Lett.* **39**, 715-718 (2018).
8. Cai, Z. et al. Ultralow-supersaturation AL pretreatment toward low dislocation density and low radio frequency loss GaN/AlN Epi-stacks on high-resistivity Si substrates. *ACS Appl. Electron. Mater.* **4**, 4113-4118 (2022).
9. Sha, W. et al. Enhanced photoluminescence of flexible InGaN/GaN multiple quantum wells on fabric by piezo-phototronic effect. *ACS Appl. Mater. Interfaces* **14**, 3000-3007 (2022).
10. Oh, J.-T. et al. High-performance GaN-based light emitting diodes grown on 8-inch Si substrate by using a combined low-temperature and high-temperature-grown AlN buffer layer. *J. Alloys Compd.* **732**, 630-636 (2018).
11. Wang, K., Li, M., Yang, Z., Wu, J. & Yu, T. Stress control and dislocation reduction in the initial growth of GaN on Si (111) substrates by using a thin GaN transition layer. *CrystEngComm* **21**, 4792-4797 (2019).
12. Liu, S. et al. Drive High Power UVC‐LED Wafer into Low‐Cost 4‐Inch Era: Effect of Strain Modulation. *Adv. Funct. Mater.* **32**, 2112111 (2022).
13. Lee, K. et al. Optical characteristics of InGaN/GaN light-emitting diodes depending on wafer bowing controlled by laser-treated grid patterns. *Opt. Express* **24**, 24153-24160 (2016).
14. Zhang, K. et al. in *SID Symp. Dig. Tech. Pap.* 1764-1767 (Wiley Online Library).
15. Lee, S. et al. Fabrication of Less Bowed Light-Emitting Diodes on Sapphire Substrates with a SiO 2 Thin Film on Their Back Sides. *Journal of the Korean Physical Society* **75**, 480-484 (2019).
16. Huang, C.-C. et al. in *2018 IEEE 2nd Electron Devices Technology and Manufacturing Conference (EDTM).* 257-259 (IEEE).\
17. Bae, J. et al. Quantum dot-integrated GaN light-emitting diodes with resolution beyond the retinal limit. *Nat. Commun.* **13**, 1862 (2022).
18. Nishikawa, A., Loesing, A. & Slischka, B. in *SID Symp. Dig. Tech. Pap.* 591-594 (Wiley Online Library).
19. Lee, H.-P., Perozek, J., Rosario, L. & Bayram, C. Investigation of AlGaN/GaN high electron mobility transistor structures on 200-mm silicon (111) substrates employing different buffer layer configurations. *Sci. Rep.* **6**, 37588 (2016).
20. Cheng, J. et al. Growth of high quality and uniformity AlGaN/GaN heterostructures on Si substrates using a single AlGaN layer with low Al composition. *Sci. Rep.* **6**, 23020 (2016).
21. Lin, P.-J. et al. Controlling the stress of growing GaN on 150-mm Si (111) in an AlN/GaN strained layer superlattice. *Appl. Surf. Sci.* **362**, 434-440 (2016).
22. Ji, X. et al. 3400 PPI Active-Matrix Monolithic Blue and Green Micro-LED Display. *IEEE Trans. Electron Devices* **70**, 4689-4693 (2023).
23. Yu, J. et al. Gallium Nitride Blue/Green Micro-LEDs for High Brightness and Transparency Display. *IEEE Electron Device Lett.* **44**, 281-284 (2022).
24. Shin, J. et al. Vertical full-colour micro-LEDs via 2D materials-based layer transfer. *Nature* **614**, 81-87 (2023).
25. Day, J. et al. III-Nitride full-scale high-resolution microdisplays. *Appl. Phys. Lett.* **99**, 031116 (2011).
26. Liu, Y. et al. Analysis of size dependence and the behavior under ultrahigh current density injection condition of GaN-based Micro-LEDs with pixel size down to 3 μm. *J. Phys. D: Appl. Phys.* **55**, 315107 (2022).
27. Liu, Y., Zhang, K., Hyun, B.-R., Kwok, H. S. & Liu, Z. High-brightness InGaN/GaN micro-LEDs with secondary peak effect for displays. *IEEE Electron Device Lett.* **41**, 1380-1383 (2020).
28. Zhang, L., Ou, F., Chong, W. C., Chen, Y. & Li, Q. Wafer‐scale monolithic hybrid integration of Si‐based IC and III–V epi‐layers—A mass manufacturable approach for active matrix micro‐LED micro‐displays. *J. Soc. Inf. Disp.* **26**, 137-145 (2018).
29. Templier, F. GaN‐based emissive microdisplays: a very promising technology for compact, ultra‐high brightness display systems. *J. Soc. Inf. Disp.* **24**, 669-675 (2016).
